# Correspondence

**Published:** 1876-04

**Authors:** J. H. Etheridge

**Affiliations:** No. 240 Wabash Av., Secretary Medical Press Association


### Correspondence.

The following contribution to the Library of the Chicago Medical Press Association from Dr. G. H. Tebo, Mt. Sterling, Ill., is hereby acknowledged with great pleasure: Eberle's Therapeutics, 1842. Graves' Clinical Lectures, 1842. Good's Study of Medicine, 1836. Graham's Chemistry, 1843. The Northwestern Medical and Surg. Journal, 1851, 1852, 1853, 1856. The Chicago Med. Journal, 1859, 1860. The American Journal Med. Sciences, 1854, 1855, 1856, 1857. The Medical News and Library, 1869. Seventy pamphlets and journals on various topics.

The officers of this Association wish to keep constantly before the profession the fact that any and all books, journals, pamphlets, monograms, etc., on medicine will be carefully preserved for reference, and that it is their earnest desire to complete sets of periodicals, such as are mentioned above. The consummation of this desire will give to the metropolis of the West the most complete medical library this side of the Atlantic cities. Every regular physician in Chicago and elsewhere being eligible to membership in this Association or to admission to the books of the library, is most cordially invited to consider the invitation to send books and journals to the undersigned (by express at his expense) for the Association, a personal matter, and to forthwith contribute all the old medical literature which he has practically cast aside or is to consign to the waste-paper basket or the flames. Every year a great deal of material, valuable in the forming of the contemplated library—worthless taken alone—is destroyed; *such* material is what is wanted. Many physicians have full years of journals tied up and stored away on the top shelves of their book cases; these bundles are never r[illegible]ed or referred to. They also have old text books [illegible]ch they never refer to, in these latter days of bette[illegible]ks. They also have many a bundle of old printed lectures and pamphlets, whose titles even they've forgotten long ago. All such literature, useless to the owners, this Association will prize highly and will thankfully receive. Gentlemen, will you not take down such books and ship them to Chicago where they will be preserved, and you thus become contributors to one of the grandest libraries in our country?

J. H. ETHERIDGE, M.D.,

No. 240 Wabash Av.,

*Secretary Medical Press Association.*

